# Clinical characteristics and management outcomes of pediatric patients with Coats disease complicated with retinoschisis (retinal cyst)

**DOI:** 10.3389/fmed.2025.1369479

**Published:** 2025-02-05

**Authors:** Tingyi Liang, Shaochi Zhang, Xiang Zhang, Xiaohan Wang, Xunda Ji, Peiquan Zhao

**Affiliations:** ^1^Department of Ophthalmology, Xinhua Hospital Affiliated to Shanghai Jiao Tong University School of Medicine, Shanghai, China; ^2^Department of Ophthalmology, Ningxia Eye Hospital, People's Hospital of Ningxia Hui Autonomous Region, Yinchuan, China

**Keywords:** Coats disease, retinoschisis, retinal cyst, clinical characteristics, surgical management

## Abstract

**Purpose:**

To present the clinical characteristics and management outcomes of Coats disease with retinoschisis (retinal cyst).

**Methods:**

This was a retrospective review of 13 eyes from 13 consecutive Coats disease patients with retinal cyst. Clinical findings from multimodal ophthalmic imaging were recorded to characterize retinal cyst in Coats disease. All eyes were initially treated with endolaser photocoagulation combined with external drainage of cystic fluid and intravitreal ranibizumab injection. Patients were subsequently followed up to analyze anatomic and visual outcomes.

**Results:**

All retinal cysts were associated with extensive retinal exudation; 3 eyes (23.1%) presented with exudative retinal detachment. A total of 92.3% (12/13) of the retinal cysts were located in the inferotemporal quadrant. Fluorescein angiography demonstrated telangiectasia surrounding and/or at the edge of the retinal cyst. Eleven eyes (84.6%) underwent endolaser photocoagulation via a nonvitrectomy approach, and 2 eyes (15.4%) underwent endolaser photocoagulation after vitrectomy due to the presence of epiretinal membrane. An average of 1.5 additional laser photocoagulation sessions was performed per patient. After a median follow-up of 23 months (range, 11–33 months), all eyes demonstrated retinal cyst resolution and no active disease. Subretinal fibrosis was observed in 9 eyes (69.2%); of these, 8 had macular subretinal nodules. In total, 30.8% of the eyes had a final vision ≥20/200.

**Conclusion:**

The presence of retinoschisis (retinal cyst) in Coats disease is strongly associated with retinal telangiectasia and exudation and is indicative of active vascular disease requiring effective ablative treatment. We propose a novel approach, endolaser photocoagulation combined with external drainage of cystic fluid and intravitreal ranibizumab injection, which was effective in achieving final anatomic improvement with retinal cyst resolution.

## Introduction

Coats disease is a rare idiopathic retinal vascular abnormality that commonly affects the unilateral eye in young males. Coats disease occurs relatively infrequently in female population but has a higher rate of bilateral involvement (1). Coats disease is typically characterized by retinal telangiectasia and exudation and the subsequent development of exudative retinal detachment (ERD) (2). Destruction of the retinal vascular structure is an important pathological change in Coats disease that results in blood–retinal barrier damage, increased vascular permeability, and ultimately leakage of a lipid-rich exudate into the retina (3).

Shields et al. (4) classified Coats disease into 5 stages: Stage 1: retinal telangiectasia; Stage 2: telangiectasia and exudation (2A: extrafoveal exudation, 2B: foveal exudation); Stage 3: ERD (3A: subtotal RD, 3B: total RD); Stage 4: total RD and neovascular glaucoma; and Stage 5: end stage. Treatment choice is based on the disease stage in Coats disease. However, regardless of the disease stage, the basic principle in the treatment of Coats disease is ablation of telangiectatic vessels, resolution of subretinal exudation, and prevention of disease progression. Clinically, the most commonly used and effective ablative treatment for Coats disease is laser photocoagulation, even in advanced stages (5, 6). In the early stages (Stage 1 and Stage 2), the disease is easily controlled by laser photocoagulation or cryotherapy. However, management of advanced Stage 3 Coats disease with ERD is tricky (7). Especially for Stage 3B Coats disease with total ERD, external drainage of subretinal fluid is preferred to fascinate subsequent ablative treatment (8). In severe cases, vitreoretinal surgery is considered. We previously reported a novel endolaser photocoagulation technique via a nonvitrectomy approach for treating advanced Coats disease; this technique has been shown to be less invasive than traditional methods while effectively ablating telangiectatic vessels (9, 10). For Stage 4 Coats disease, anti-glaucoma surgery is required in addition to conventional ablative treatment. Observation or enucleation is considered in Stage 5 Coats disease.

Retinoschisis in Coats disease, also known as retinal cyst, refers to an exudative fluid-filled cavity accompanied by splitting of the neurosensory retina (11). The development of retinoschisis in Coats disease may be related to prolonged vascular leakage, retinal exudation, and the resulting retinal degeneration (12, 13). The occurrence of retinoschisis in Coats disease may indicate a vasoactive state, which requires more aggressive and effective treatment (14, 15). Due to the rarity of this condition, herein, we report the clinical characteristics and surgical management of a case series of pediatric patients with Coats disease complicated with retinoschisis (retinal cyst).

## Methods

This was a retrospective consecutive case series review of 13 pediatric patients (13 eyes) with Coats disease complicated with retinal cysts treated at Xinhua Hospital Affiliated to Shanghai Jiao Tong University School of Medicine between November 2018 and April 2022. Patients were excluded if they had a history of other ocular or systemic disease. The study adhered to the tenets of the Declaration of Helsinki and was approved by the Ethics Committee of Xinhua Hospital Affiliated to Shanghai Jiao Tong University School of Medicine. Written informed consent was obtained from each patient and their guardians.

Clinical information, including age, sex, eye laterality, medical history, and visual acuity, was collected from each patient. Patients underwent comprehensive ophthalmic examinations. The anterior segments were examined via slit-lamp microscopy. Fundus examinations were performed via indirect ophthalmoscopy with a + 20 D lens. Wide-field color fundus images were taken using RetCam III (Clarity Medical Systems, Pleasanton, CA, USA) or Optos Optomap 200Tx (Optos, Dunfermline, Scotland). Fluorescein angiography (FA) using RetCam III or Heidelberg Spectralis (Heidelberg Engineering, Heidelberg, Germany) was used to evaluate the area of retinal telangiectatic vessels and nonperfusion areas. Ultrasonography (Aviso, Quantel Medical, Clermont-Ferrand, France) and optical coherence tomography (OCT) (Optovue, Fremont, CA, USA) were used to evaluate the presence of retinal detachment and retinoschisis.

### Surgical management

In our study, all treated eyes underwent endolaser photocoagulation to ablate retinal telangiectatic vessels and nonperfusion areas according to the FA findings. All surgical procedures were performed by the same experienced surgeon (Peiquan Zhao). In eyes with a normal vitreous status, endolaser photocoagulation was performed via a two-port pars plana nonvitrectomy approach. The entire retinal telangiectatic vessels were examined through visualization with a noncontact wide-angle viewing system and then directly ablated with a 532-nm green endolaser. Vitrectomy combined with endolaser photocoagulation was performed for patients presenting with epiretinal membrane, vitreoretinal traction, or vitreous hemorrhage. During the treatment course, the treated eyes underwent a transscleral puncture procedure to drain cystic fluid and flatten the retina. The retinal cysts were transsclerally punctured using a modified 5-mL syringe (Becton, Dickinson and Company, New Jersey, USA) with approximately one-third of the needle tip bent at 150 degrees. The puncture entry site was localized by sliding the blunt side of the syringe needle and depressing the sclera under the surgical microscope. After confirming the location of the puncture, the sharp side of the syringe needle was perpendicularly and gradually inserted into the cystic cavity to drain cystic fluid. Subsequently, as the retina flattened, complete laser ablation of the telangiectatic vessels around the cyst was performed. In addition, an intravitreal injection of ranibizumab (0.5 mg/0.05 mL) was administered. Supplementary Video S1 shows the detailed surgical procedures, including the method for making a modified transscleral puncture needle.

Patients were followed up postoperatively at 1 week, 1 month, and then at 3- to 6-month intervals. The treated eyes were observed for retinal cyst and retinal telangiectatic vessel resolution. After the initial treatment, laser ablation was repeated if the retinal telangiectatic vessels did not completely regress or recurred. Complications, including hemorrhage, iatrogenic retinal damage, and vitreoretinal fibrosis, were evaluated.

## Results

A total of 13 pediatric patients with Coats disease complicated with retinal cysts were included in this study (Table 1). The average age at presentation was 5.5 years (median 6 years; range 1–12 years), and the predominant sex was male (92.3%). All patients had unilateral disease. The mean follow-up period was 22 months (median 23 months; range 11–33 months). Supplementary Table S1 shows the detailed follow-up results of this study.

**Table 1:** Clinical characteristics of the patients.

Patient	Sex/age (years)/eye laterality	Stage	Location of retinal cyst	Treatment history	Initial VA	Initial treatment	Additional laser photocoagulation (No.)	Types of fibrosis present	Final VA	Follow-up (months)
1	M/12/R	2B	IT	/	CF	EL + XD + IVR	1	SR, MN	20/400	24
2	M/1/L	2B	IT	/	NA	EL + XD + IVR	1	/	NA	23
3	M/7/L	2B	IT	LIO + IVR	CF	EL + XD + IVR	1	SR, MN	20/400	33
4	M/8/L	2B	IT	/	NA	EL + XD + IVR	2	SR, MN	CF	23
5	M/5/R	2B	IT	/	20/400	EL + XD + IVR	2	SR, MN	20/200	22
6	M/3/R	3A	IT	/	CF	EL + XD + IVR	2	SR	20/600	21
7	F/6/R	3A	IT	/	20/200	EL + XD + IVR	2	SR, MN	20/200	26
8	M/3/R	2B	IT	LIO + IVR	NA	EL + XD + IVR	1	/	NA	27
9	M/7/R	3A	ST	IVR	LP	PPV + EL + XD + IVR	1	/	HM	25
10	M/5/L	2B	IT	/	20/200	EL + XD + IVR	1	/	20/150	14
11	M/6/R	2B	IT	LIO + IVR	HM	PPV + EL + XD + IVR	1	SR, MN	CF	18
12	M/6/R	2B	IT	/	20/200	EL + XD + IVR	2	SR, MN	20/150	20
13	M/3/L	2B	IT	/	NA	EL + XD + IVR	3	SR, MN	CF	11

IT, inferior-temporal quadrant; ST, superior-temporal quadrant; LIO, laser indirect ophthalmoscopy; IVR, intravitreal injection of ranibizumab; VA, visual acuity; CF, counting fingers; HM, hand motion; NA, not applicable; LP, light perception; EL, endolaser photocoagulation; XD, external drainage of cystic fluid; PPV, pars plana vitrectomy; SR, subretinal fibrosis, MV, macular nodule.

**Figure 1:**
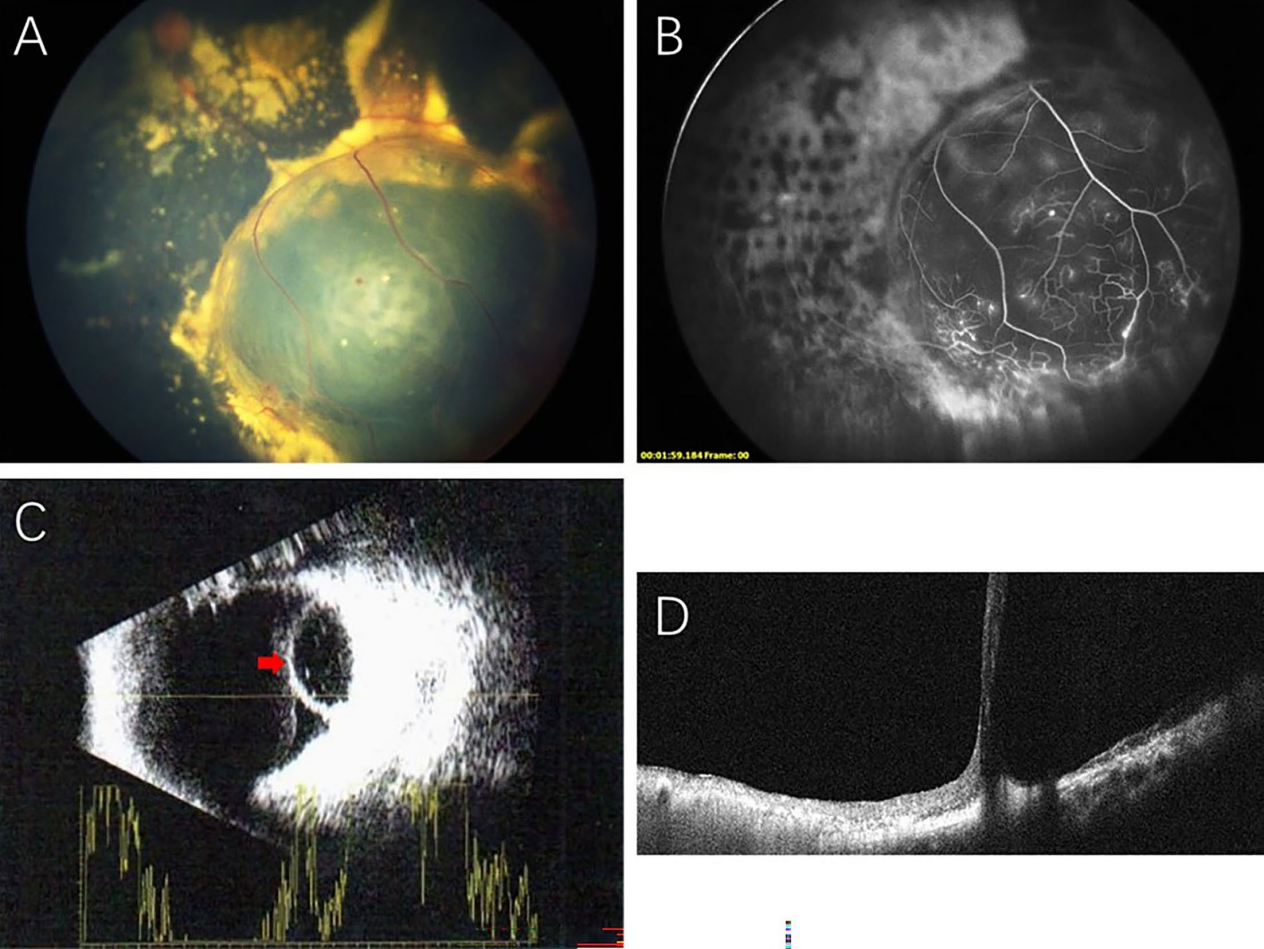
Multimodal imaging showing retinal cyst in Coats disease: **(A)** A massive retinal cyst in the inferotemporal retina surrounded by dense subretinal exudation. **(B)** FA showing nonperfusion areas over the retinal cyst and retinal telangiectatic vessels at the edge of the retinal cyst. **(C)** B-scan ultrasonography showing an intraocular cystic mass with a hyperechoic area in the anterior ocular wall (red arrow). **(D)** OCT showing a retinal cystic cavity with splitting of the neurosensory retina.

In our study, the retinal cysts of the Coats disease patients manifested as spherical, well-circumscribed, protruding macrocysts located in the peripheral retina (anterior to the vortex veins). The retinal cysts were mostly located in the inferotemporal quadrant (12/13, 92.3%), and only one case was located in the superotemporal quadrant. All eyes demonstrated extensive retinal exudation with macular involvement, and the retinal cysts were localized within areas of retinal exudation. However, ERD was observed in only 3 eyes (23.1%). Correspondingly, 10 of the treated eyes (76.9%) were classified as Stage 2B, and 3 (23.1%) were classified as Stage 3A. FA showed nonperfusion areas over the retinal cysts and typical retinal telangiectatic vessels surrounding and/or at the edges of the retinal cysts. Ultrasonography and OCT confirmed the presence of retinoschisis and retinal cystic cavity (Figure 1).

**Figure 2:**
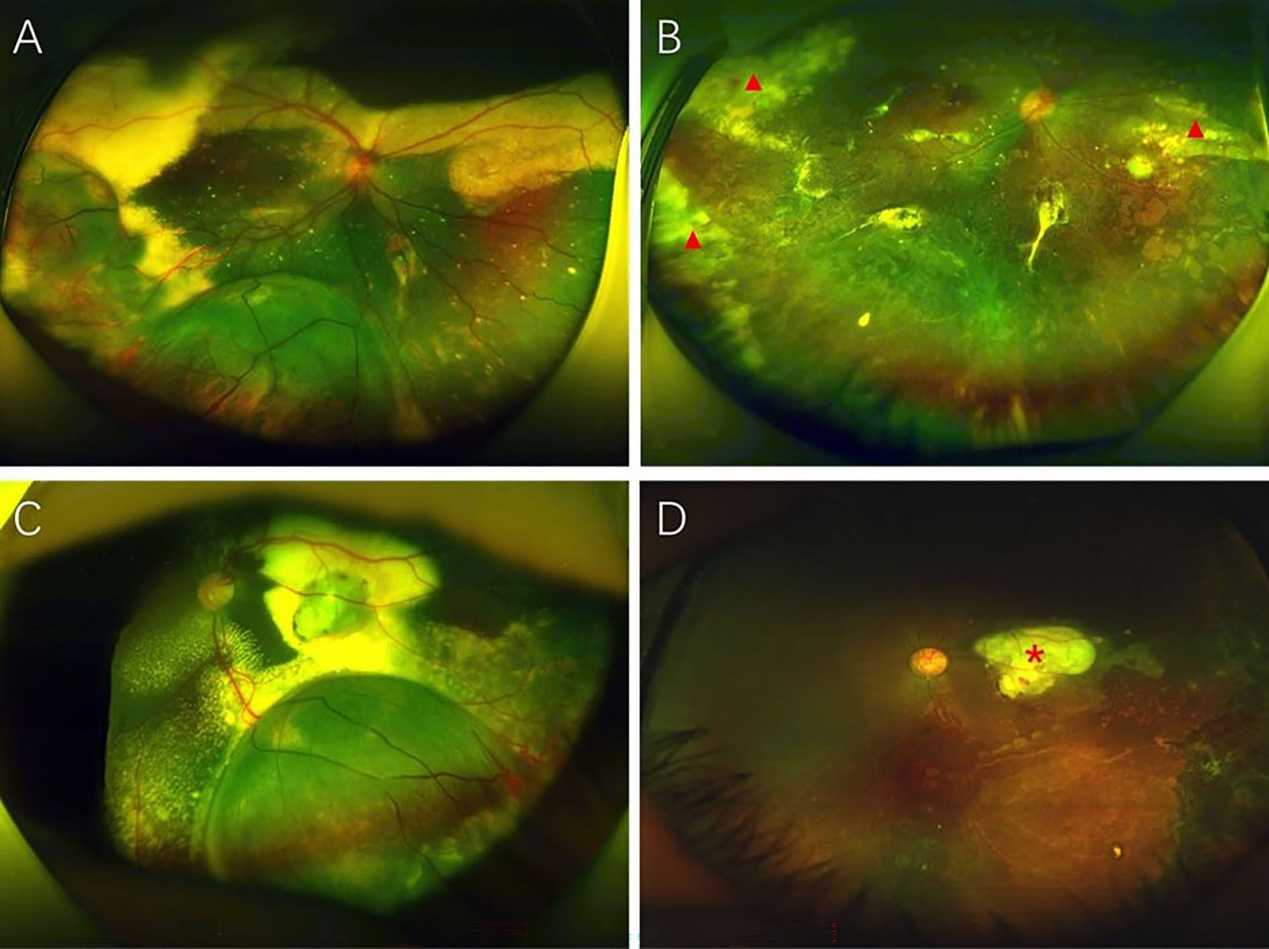
An 8-year-old boy (case 4) with Coats disease presenting with retinal cysts. The patient had extensive retinal telangiectasia and exudation with retinal cysts in the temporal retina **(A)**. After endolaser ablative treatment combined with external drainage of cystic fluid, intravitreal ranibizumab injection and 2 additional laser photocoagulation treatments, the retinal cystic cavities resolved with no active disease **(B)**. Peripheral subretinal fibrosis (red triangles) was observed and remained stable. A 3-year-old boy (case 6) with Coats disease presenting with a retinal cyst. Preoperative fundus photography demonstrated a massive retinal cyst surrounded by retinal telangiectasia and exudation **(C)**. The patient was initially treated with endolaser photocoagulation combined with external drainage of cystic fluid, an intravitreal injection of ranibizumab and 2 additional laser photocoagulation treatments. Final anatomic improvement with retinal cyst resolution was observed **(D)**. Macular subretinal nodules were observed (red asterisk).

Before referral to our hospital, 4 patients had a treatment history: 3 patients had undergone laser photocoagulation combined with intravitreal ranibizumab injections, and 1 patient had received an intravitreal ranibizumab injection. In the present study, all eyes underwent endolaser photocoagulation to ablate the retinal telangiectatic vessels and nonperfusion areas. Eleven eyes (84.6%) underwent endolaser photocoagulation via a two-port nonvitrectomy approach, and 2 eyes (15.4%) underwent endolaser photocoagulation combined with vitrectomy due to the presence of epiretinal membrane. During treatment, transscleral puncture of the retinal cyst was successfully performed in all eyes. In one eye (case 5), localized minimal subretinal hemorrhage was observed at the puncture site. Bleeding was stopped by temporarily pressing the eyeball to increase the intraocular pressure, and the subretinal hemorrhage was resolved 1 month later. No other serious complications occurred, such as iatrogenic retinal damage or retinal detachment. After the initial treatment, the treated eyes received 1 to 3 (mean 1.5 sessions) additional laser photocoagulation treatments to completely eliminate the telangiectatic vessels. At the last visit, the treated eyes demonstrated retinal cyst resolution; moreover, none of the eyes demonstrated disease progression or active telangiectatic vessels (Figure 2). Subretinal fibrosis was observed in 9 eyes (69.2%), including 8 eyes (61.5%) with macular subretinal nodules.

In terms of visual outcomes, among the 9 patients (69.2%) who cooperated with the vision examination, 7 (77.8%) experienced vision improvement; however, only 4 of the 13 eyes (30.8%) had a final vision greater than or equal to 20/200.

## Discussion

Coats disease is a retinal vascular disorder characterized by retinal telangiectasia and vascular leakage with progressive retinal exudation and ERD (16). Retinoschisis, also called retinal cyst in Coats disease, refers to the splitting of the neurosensory layer of the retina. Shields et al. reported that the incidence of retinal cysts was 11% in a case series of 150 patients with Coats disease; however, the specific clinical characteristics were not described (2). In the present study, we reported the clinical and treatment features of pediatric patients with Coats disease complicated with retinal cysts.

Retinal cyst, a kind of secondary retinoschisis, is related to chronic retinal detachment due to hypoxia and dysmetabolism of retinal pigment epithelial cells (13, 17–19). However, in our clinical series, ERD was observed in only three patients. The retinal cysts were typically localized within areas of prominent retinal telangiectasia and massive subretinal exudation. Scott et al. reported that 100% of patients with Coats disease with retinoschisis and macrocysts had dense subretinal exudation, but only 38.8% had exudative retinal detachment (14). Liu et al. reported that Coats disease patients with retinal cysts presented with more clock-hours of telangiectasia (15). These findings suggest that retinal cysts in patients with Coats disease might be more strongly associated with retinal telangiectasia and exudation than with retinal detachment.

In other aspects, our case series showed that the retinal cysts were mostly located in the inferotemporal quadrant (92.3%). This is due to the effects of gravity, which caused subretinal fluid to preferentially accumulate in the inferior quadrant. More importantly, it is also associated with the location of the retinal telangiectatic vessels, which are located mainly in the peripheral retina. Vascular abnormalities and leakage play important roles in the formation of retinal cysts in Coats disease, which can be confirmed by the FA findings of the networks of telangiectatic vasculature surrounding and/or at the edges of the retinal cysts.

The presence of retinal cysts in patients with Coats disease implies active vascular disease, which may require more aggressive ablative treatment to achieve disease control. Laser photocoagulation of retinal telangiectasia and nonperfusion areas is the most commonly used and effective ablative treatment for active Coats disease (20). Effective laser ablative treatment can promote subretinal exudation and fluid resolution and inhibit neovascularization (5, 6). In the present study, we preferred endolaser photocoagulation via a nonvitrectomy approach for ablating telangiectatic vessels, as we have previously reported that this approach is more efficient than traditional methods for ablating telangiectasia and reducing the need for repetitive treatment sessions (9, 10). Vitrectomy followed by endolaser photocoagulation was considered for patients presenting with vitreoretinal disease, such as vitreous hemorrhage and vitreoretinal traction. Notably, a prominently raised retinal cyst made it difficult to perform laser photocoagulation of the telangiectatic vessels on the surface and edge of the cyst; thus, we performed a transscleral puncture of the retinal cyst to drain cystic fluid and flatten the retina. Subsequently, complete laser ablation of retinal telangiectasia was performed. Anti-vascular endothelial growth factor (anti-VEGF) treatment is considered an adjuvant to ablative treatment for Coats disease and can facilitate the resolution of subretinal fluid and exudation and control disease progression (21–23).

In the present study, all the treated eyes demonstrated retinal cyst resolution and no active telangiectatic vessels. Final anatomic success can be attributed to effective ablative treatment. In our opinion, compared with conventional repetitive laser photocoagulation, the proposed endolaser photocoagulation approach can directly target telangiectatic vessels, enhancing the thermal effect of the laser while avoiding energy loss. Moreover, transscleral puncture of retinal cysts and flattening of the retina facilitated complete ablation of the retinal telangiectatic vessels. After the initial endolaser ablative treatment, the treated eyes received an average of 1.5 additional laser photocoagulation treatments, which is lower than the number reported in previous studies on pediatric patients with Coats disease complicated with retinal cysts. In addition, during the follow-up, we did not observe disease progression or serious complications that required additional surgical intervention. In a case series (23 eyes) of Coats disease patients with retinal cysts, Liu et al. reported that after a mean of 4.08 treatment sessions with argon laser photocoagulation, 8 eyes (35%) needed additional vitrectomy and/or cryotherapy; final anatomic improvement was achieved in 18 eyes (78.26%) (15). A similar study from Scott et al. described the management outcomes of retinal macrocysts in 18 patients with Coats disease. All patients were treated with angiography-guided photocoagulation for an average of 4.8 treatment sessions. Seven patients (39%) needed revisional intraocular surgery (vitrectomy, scleral buckling, and/or silicone oil), and 5 patients failed to achieve anatomic improvement—including one patient requiring enucleation (14). To date, the exact pathogenesis of retinoschisis in Coats disease has not been determined, which may make management difficult. Notably, in the initial treatment stage, effective ablation of retinal telangiectasia strongly affects the overall disease prognosis.

Fibrosis is frequently observed in Coats disease, but it is not yet clear whether fibrotic changes occur as a result of the natural course of Coats disease or if therapies such as anti-VEGF treatment accelerate its development. Munier et al. reported that the presence of extramacular fibrosis in Coats disease patients (69 eyes) was not associated with anti-VEGF treatment or ablative treatment but with the extent of retinal exudation and exudative retinal detachment (24). As in our study, all eyes initially demonstrated extensive retinal exudation with macular involvement; of these, 69.2% of the eyes developed subretinal fibrosis, which tended to be stable without further intervention. Moreover, the presence of fibrosis, especially macular fibrosis (nodules), portends a worse visual outcome. As a result, in our study, although vision improvement was observed in 77.8% of the cooperative patients, an unsatisfactory final visual status was closely related to the occurrence of macular subretinal fibrosis.

The present study has several limitations. First, this study had a relatively short follow-up. Extended serial follow-up will be valuable for assessing long-term anatomic and visual outcomes. Second, similar to many previously published studies on Coats disease, the sample size of this study was not sufficiently large. Finally, this study lacked a control group for evaluating the true benefit of endolaser ablative treatment over conventional laser photocoagulation. However, the rarity of Coats disease, especially in the presence of retinoschisis, limits this study to a retrospective, noncomparative case series. A multicenter, randomized controlled trial for the management of Coats disease with retinal cysts will be greatly beneficial in the future.

In summary, our study reports on a case series of pediatric patients with Coats disease complicated with retinoschisis (retinal cyst) and indicates that the presence of retinal cysts in Coats disease is strongly associated with retinal telangiectasia and exudation and implies active vascular disease. There is no standard treatment for this condition; however, ablative treatment for retinal telangiectatic vessels is critical for achieving disease control and retinal cyst resolution. We provided an effective treatment strategy consisting of endolaser photocoagulation combined with external drainage of cystic fluid and intravitreal ranibizumab injection, which demonstrated final anatomic improvement with retinal cyst resolution.

## Data Availability

The original contributions presented in the study are included in the article/Supplementary material, further inquiries can be directed to the corresponding authors.

## Supplementary material

SUPPLEMENTARY VIDEO S1 Surgical video of the novel treatment approach for Coats disease complicated with retinal cyst, consisting of two-port pars plana nonvitrectomy endolaser photocoagulation, external drainage of cystic fluid and intravitreal ranibizumab injection.

SUPPLEMENTARY TABLE S1 Detailed follow-up results in the present study.
